# Alcohol and the Male Reproductive System

**Published:** 2001

**Authors:** Mary Ann Emanuele, Nicholas Emanuele

**Affiliations:** Mary Ann Emanuele, M.D., is a professor in the Department of Medicine, the Department of Molecular and Cellular Biochemistry, and the Division of Research on Drugs of Abuse, Loyola University Stritch School of Medicine, Maywood, Illinois. Nicholas V. Emanuele, M.D., is a professor in the Department of Medicine, the Division of Research on Drugs of Abuse, Loyola University Stritch School of Medicine, Maywood, Illinois, and a staff physician at the Veterans Affairs Hospital, Hines, Illinois

**Keywords:** hypothalamic-pituitary-gonadal axis, reproductive effects of AODU (alcohol and other drug use), male, reproductive system, testicles, nitric oxide, oxidation, ethanol-to-acetaldehyde metabolism, apoptosis, luteinizing hormone-releasing hormone, fertility, opioids

## Abstract

Alcohol use affects all three parts of the hypothalamic-pituitary-gonadal (HPG) axis, a system of endocrine glands and hormones involved in male reproduction. Alcohol use is associated with low testosterone and altered levels of additional reproductive hormones. Researchers are investigating several potential mechanisms for alcohol’s damage. These mechanisms are related to alcohol metabolism, alcohol-related cell damage, and other hormonal reactions associated with alcohol consumption. Chronic alcohol use in male rats also has been shown to affect their reproductive ability and the health of their offspring.

The endocrine system, which is made up of several hormone-producing organs throughout the body, is integral to all normal body functions, including growth, development, metabolism, and reproduction. This article reviews research on the effect of alcohol use on the part of the endocrine system involved in male reproduction, the hypothalamic-pituitary-gonadal (HPG) axis. This system of endocrine glands and hormones includes a brain region called the hypothalamus; the pituitary gland, located at the base of the brain; and the male gonads (testes). The article also highlights promising new strategies for preventing or reversing alcohol’s harmful effects on the male reproductive system and describes research investigating the molecular mechanisms by which alcohol acts on this system.

## Overview of the Male Reproductive System

Of the three components of the HPG axis, the hypothalamus and the pituitary gland have solely regulatory functions, which are mediated by the hormones they produce and secrete, as described in the next paragraph. The third component—the testes—also produces key hormones, including testosterone, which control male sexual characteristics and behaviors. In addition, the testes are responsible for sperm production.

**Figure 1:**
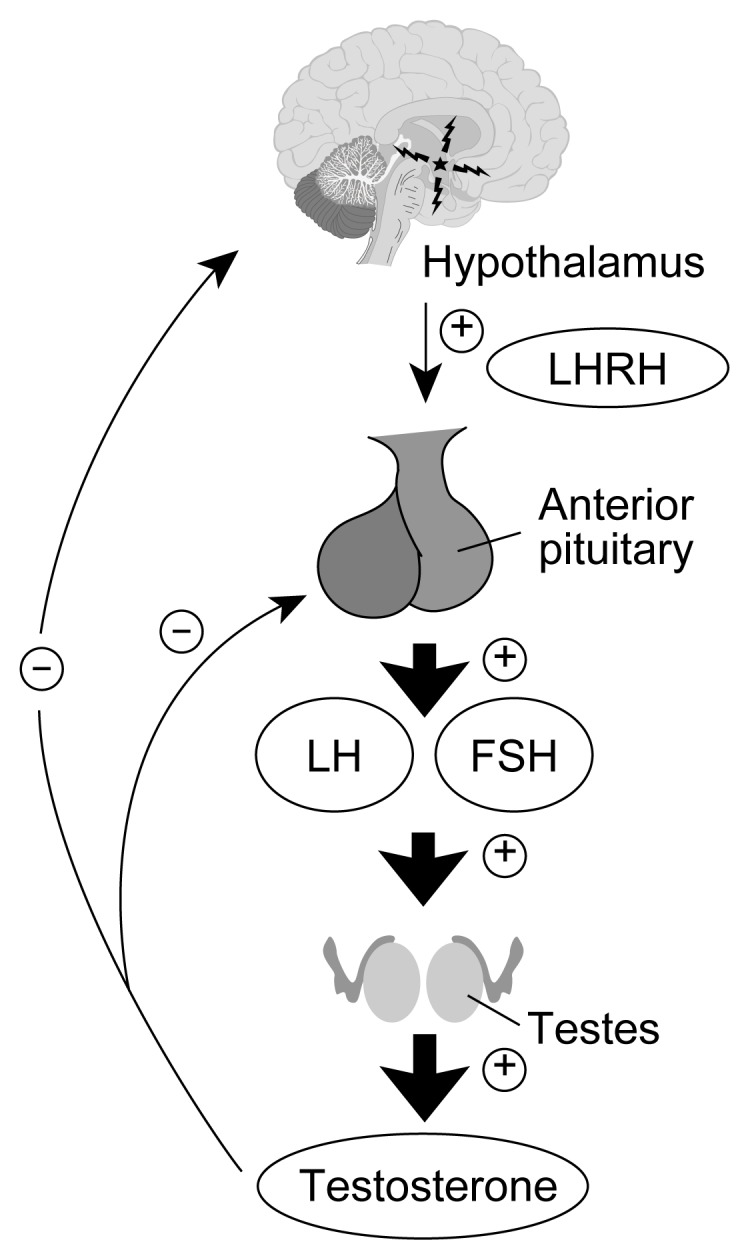
The hypothalamic-pituitary-gonadal axis. The hypothalamus produces luteinizing hormone releasing hormone (LHRH), which is released to the pituitary gland. In response to the LHRH signal, the pituitary gland produces luteinizing hormone (LH) and follicle-stimulating hormone (FSH). In males, LH stimulates testosterone production and FSH is important to sperm maturation. Testosterone circulates in the blood back to the hypothalamic-pituitary unit and regulates the further production and secretion of LHRH and LH. NOTE: ⊕ = stimulatory effect; ⊖ = inhibitory effect.

The hypothalamus produces luteinizing hormone-releasing hormone (LHRH), which is released in pulses into a system of blood vessels that connect the hypothalamus and the pituitary gland. In response to the LHRH signal, the pituitary gland produces two protein hormones called gonadotropins. These two gonadotropin hormones—luteinizing hormone (LH) and follicle-stimulating hormone (FSH)—are then released into the body’s general circulation and act primarily at the level of the gonads. In males, LH stimulates testosterone production from specialized cells called Leydig cells. FSH is important to sperm maturation in another compartment of the testes, the epididymis. Testosterone circulates in the blood back to the hypothalamic-pituitary unit and regulates the further production and secretion of LHRH and LH (see figure 1). When the system is functioning normally, a low testosterone level results in a rise in pituitary gonadotropins. Prolactin, a third reproductive hormone synthesized in the pituitary gland, is important to normal LHRH synthesis and secretion.

Low levels of testosterone (i.e., hypogonadism) in adult men have been associated with a variety of medical problems including accelerated osteoporosis, decreased muscle and prostate function, anemia, altered immune function, and decreased reproductive ability (Klein and Duwall 1994; Jackson and Klerekoper 1990; Azad et al. 1991; Berczi et al. 1981; Hadley 1988). Each of these conditions can cause significant health problems. These effects of low testosterone are greater in adult men who have had low testosterone levels since adolescence compared with adult men who experience diminished testosterone levels only in adulthood (Hadley 1988; Yen and Jaffe 1991). An adolescent or teenager who experiences short-term, intermittent decreases in testosterone or permanent hypogonadism is predisposed to experience these problems later in life.

Research with animals has consistently demonstrated an association between both acute (i.e., one time, one occasion) and chronic (i.e., long-term) alcohol consumption and low testosterone. As testosterone levels decrease, levels of LH and FSH would be expected to increase to stimulate the production of more testosterone. However, studies with young (i.e., pubertal) male rats indicate that both acute and chronic alcohol exposure result in profound testosterone suppression accompanied by lower or normal LH and FSH levels, when elevated levels are expected (Hadley 1988; Yen and Jaffe 1991). This suggests that the hypothalamic cells which produce LHRH do not function correctly when the feedback normally provided by testosterone is removed (i.e., when testosterone levels decrease). Thus it appears that alcohol’s damaging effects on reproduction are mediated at all three levels of the male reproductive unit: the hypothalamus, pituitary, and testes.

## Alcohol and the Testes

**Figure 2:**
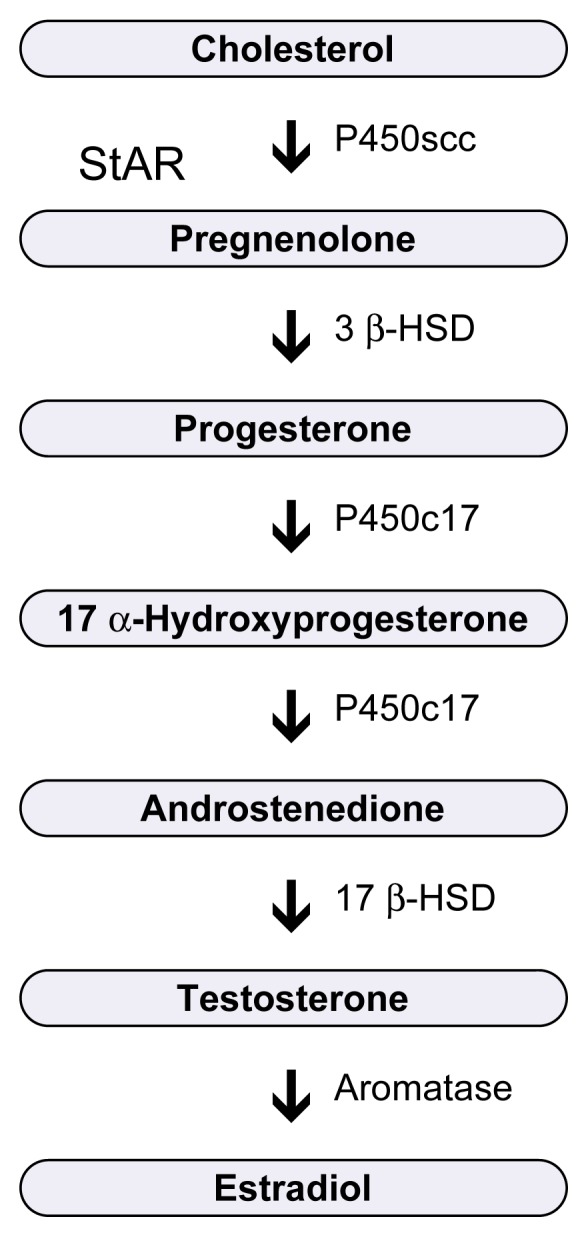
Testosterone biosynthetic pathway. Multiple enzymes are necessary to synthesize testosterone. These are shown to the right of the arrows. The arrows indicate the different steroid precursors of testosterone that are synthesized at each step. StAR = steroidogenic acute regulatory protein

Most studies of alcohol’s effects on male reproduction have been conducted in rats because the rat model mimics the human male reproductive system. Research has demonstrated that both acute and chronic alcohol exposure are associated with low levels of hypothalamic LHRH and pituitary LH in the adult (Cicero 1982; Salonen et al. 1992) and pubertal male rat, and further studies have suggested that alcohol inhibits testosterone secretion by the testes as well (Little et al. 1992). For example, several studies have examined alcohol’s effects on testosterone synthesis, which occurs via the testosterone biosynthetic pathway. This pathway consists of a series of steroid precursors of testosterone and the respective enzymes necessary to synthesize each precursor from the previous one (Hadley 1988) (see figure 2). For several reasons related to differences in experimental design, it is not possible to conclude precisely where alcohol acts in the testosterone synthetic pathway, although it seems likely that more than one site of action exists. Researchers currently are investigating alcohol’s effects on both the molecular steps integral to manufacturing the enzymes in the testosterone pathway and the steroid precursors of testosterone. Researchers also are exploring strategies to prevent alcohol’s suppressive effects on testosterone synthesis. One such strategy involves giving male rats testosterone pellets to replace testosterone; other methods involve blocking the degradation of testosterone with aromatase inhibitors (aromatase is a key enzyme involved in converting testosterone to estrogen) such as Fadrozol.

### Mechanisms of Alcohol-Induced Testicular Damage

Although it is well known that chronic alcohol abuse produces sexual dysfunction and impairs sperm production in both humans and animals (Yen and Jaffe 1991), the mechanisms of this alcohol-induced damage have not been fully explained. Several possible mechanisms are described below.

#### Opioids

Testicular opioids are messenger molecules similar to morphine that, when produced within the testes, suppress testosterone synthesis. One opioid, known as beta-endorphin, has been shown to increase with acute and chronic alcohol consumption and thus may be one link between alcohol use and testicular damage. For example, beta-endorphin produced within the testes suppresses testicular testosterone production and release (Gianoulakis 1990). Similarly, beta-endorphin produced in the hypothalamus results in decreased LHRH levels. In addition, opioids may increase programmed cell death (i.e., apoptosis) (Yin et al. 1999; Nanji and Hiller-Sturmhöfel 1997). Apoptosis at the gonadal level would result in the death of both Leydig and seminiferous cells, which are cells involved in sperm cell formation and maturation, leading not only to low testosterone but also to diminished sperm production. In both adult and pubertal male rats, treatment with the opioid antagonists (i.e., chemicals that prevent opioids from binding to their receptors in the brain) naloxone and naltrexone (ReVia^™^) has been successful in preventing alcohol-induced testosterone inhibition (Gianoulakis 1990).

#### Nitric Oxide

Another way that alcohol’s harmful effects on testosterone production have been reduced involves nitric oxide (NO), a ubiquitous gas that results in the dilation of blood vessels, or vasodilatation. NO is synthesized in the testes by a key enzyme, NO synthase (NOS), and inhibition of this enzyme by a variety of NOS inhibitors successfully prevents the decrease in testosterone associated with alcohol consumption (Adams et al. 1992). Therefore, future interventions aimed at preventing or reversing alcohol-induced gonadal suppression (i.e., reductions in sperm and testosterone production) may involve blocking NO synthesis.

#### Oxidation

The oxidation^1^ of alcohol, a process that occurs as part of alcohol metabolism, generates byproducts called oxidants that can contribute to cell damage and may play a role in alcohol-induced tissue damage in the testes. An imbalance between oxidants and antioxidants (i.e., substances that neutralize oxidation) can create oxidative stress, a state marked by continued production of oxidizing agents and escalating cell damage. Increased oxidative stress is a well-accepted mechanism of alcohol-induced tissue injury, particularly in the liver (Sies 1997; Aleynik et al. 1998; Polavarapu et al. 1998), heart, and central nervous system, and there is some information that this also occurs in the testes (Emanuele et al. 2001). Alcohol consumption may induce oxidative damage either by enhancing the production of toxic compounds called free radicals or by decreasing the levels of antioxidants.

Certain oxidants produced by alcohol metabolism are known as reactive oxygen species (ROS). These include anion superoxide, hydrogen peroxide, hydroxyl radicals, and nitrogen reactive species such as NO. The metabolism of alcohol and of acetaldehyde, which is the principle product of alcohol metabolism, produces highly toxic ROSs. Some data suggest that acetaldehyde is actually more toxic than alcohol to the production of testosterone, altering the process of testosterone production by inhibiting protein kinase C, a key enzyme in testosterone synthesis (Anderson et al. 1985; Chiao and Van Thiel 1983). Additional research has shown that men with chronic alcoholism and hypogonadism actually eliminate alcohol more rapidly, building up less acetaldehyde. Because the buildup of acetaldehyde in the body is nauseating, enhanced clearance of this byproduct could lead to reduced gastrointestinal side-effects from drinking (e.g., abdominal discomfort and vomiting) in men with low testosterone levels. This may increase the risk of developing a drinking problem, because a person who does not experience the negative gastrointestinal side-effects of drinking will be more likely to continue to drink, often in larger amounts (Vaubourdolle et al. 1991).

#### Cell Damage

Because testicular membranes are rich in molecules known as fatty acids (i.e., lipids), which are prone to oxidative injury, it is reasonable to consider that lipid peroxidation (i.e., damage to the cell membranes) may contribute to the gonadal dysfunction that occurs as a result of acute or chronic alcohol use. Peroxidation injury can be attenuated with dietary vitamin A supplementation (Sies 1997). Vitamin A, acting as an antioxidant, stabilizes testicular cell membranes by reducing lipid peroxidation and prevents the alcohol-induced atrophy that occurs in animals not receiving vitamin-A-enriched diets. Taken together, these observations suggest that the enhanced peroxidation of testicular lipids that occurs following alcohol consumption may be an important factor in the pathogenesis of alcohol-associated gonadal injury and that diets rich in vitamin A can help counteract such injury. Research currently is being conducted to examine the role of oxidative injury in alcohol-induced hypogonadism.

In addition to membrane damage, additional types of cell damage may be associated with alcohol-induced testicular damage. Heavy alcohol consumption over long periods of time results in severe cell damage that leads to cell death. Cell death occurs via two distinct mechanisms: necrosis and apoptosis. Necrosis occurs when exposure to a noxious stimulus, such as alcohol, causes the loss of the cell’s metabolic functions and damage to the cell membrane. In apoptosis, the cell actively participates in the cell death processes by activating a cascade of biochemical reactions that ultimately lead to cell shrinkage and fragmentation of the nucleus. When a cell undergoes apoptosis, the entire cell, including the nucleus, separates into numerous fragments (i.e., apoptotic bodies) (Nanji and Hiller-Sturmhöfel 1997).

In any organ, both acute and chronic alcohol exposure induce cell necrosis as well as apoptosis, and oxidative stress plays a crucial role in both processes. This also is true for the testicular germ cell (i.e., a cell important in sperm development and maturation). Oxidative injury results in the disarrangement and ultimately in the disruption of cell membranes, leading to necrotic cell death. Moreover, the oxidation of enzymes can block the metabolic processes essential for cell functioning and repair. Apoptosis is presumed to represent the last common pathway of ROS-mediated cell injury. This is induced directly by oxygen free radicals. Although germ cell apoptosis also can be triggered by various non-hormonal regulatory stimuli, including testicular toxins, heat stress, and chemotherapeutic agents, the mechanisms by which these hormonal and nonhormonal factors regulate germ cell apoptosis are not well understood (Hikim and Swerdloff 1999). Apoptosis also can be induced indirectly by an imbalance between oxidation and reduction^2^ processes in the cell and by the expression of inflammation-promoting molecules called cytokines. Ongoing work in the area of alcohol-induced apoptosis at the gonadal level will expand the knowledge in this area.

#### Other Potential Mechanisms

Other explanations for the gonadal suppression associated with alcohol involve the metabolism of alcohol to acetoacetate, a highly toxic compound, and other toxic agents that are formed from acetoacetate, such as salsolinol (Stumble et al. 1991). Similarly, alcohol may induce elevated levels of pituitary prolactin and inflammation-promoting cytokines in the brain, which may be responsible for gonadal suppression of testosterone. Disturbances in other hormonal system components that interact with the HPG axis, such as the adrenal gland, also play roles in gonadal testosterone suppression (Ogilvie and Rivier 1997). The effects of liver disease on the metabolism of gonadal steroids and circulating levels of gonadal steroids also are important to understanding alcohol-induced hypogonadism (Lieber 1994). These effects are beyond the scope of this review, however.

## Alcohol and the Male Hypothalamic-Pituitary Unit

Research on alcohol’s effects on the hypothalamus and pituitary has centered on the effects of the hormones LHRH, produced by the hypothalamus, and LH and FSH, produced by the pituitary. Whereas some studies have reported that the secretion of LHRH is reduced after acute alcohol consumption and other studies have reported no effect (see Emanuele et al. 1993), the ability of the male hypothalamus to synthesize this important hormone appears to be unaltered by alcohol at any dose (Emanuele et al. 1993).

LHRH secretion is closely regulated by a series of complex mechanisms involving various nerve impulses generated outside the hypothalamus. Alcohol could influence any of these stimuli. Multiple processes and compounds, including the opioids, lead to the activation of the LHRH pulse generator, the part of the hypothalamus responsible for LHRH secretion (Gianoulakis 1990). Other brain chemicals and nerve signals also play roles in LHRH secretion and may be adversely affected by alcohol. However, this uppermost level of the reproductive axis, the hypothalamus, seems to be the least vulnerable to the deleterious consequences of alcohol.

Researchers also have assessed the effect of alcohol on LH and FSH, the pituitary gonadotropins responsible for gonadal function. Studies in animals and humans have shown that when testosterone levels decrease LH levels do not increase as would be expected. This inability of the pituitary gland to respond appropriately to a decline in testosterone implies that alcohol has a central effect on the interaction between the nervous system and the endocrine system (Hadley 1988; Yen and Jaffe 1991).

Studies in alcohol-fed rats have established that the decrease in LH levels results from impairment in both LH production and LH secretion, and researchers have attempted to identify the specific steps in LH production and secretion that are affected by alcohol (Salonen et al. 1992; Emanuele et al. 1993). LH production is initiated by the interaction of LHRH released from the hypothalamus with specific LHRH receptors.

This interaction activates a cascade of enzymatic events in pituitary cells. Alcohol could disrupt the functioning of the LHRH receptor or its interaction with LHRH, resulting in diminished LH release. No evidence has been found to indicate that alcohol impairs the interaction of LHRH with its receptor, but alcohol may affect subsequent events. Specifically, researchers have reported that alcohol impairs the function of protein kinase C, a key enzyme in LH production, and that alcohol impairs other steps critical to LH synthesis and secretion. Additional research with rats has shown that alcohol impairs LH production by decreasing the ability of LH genetic material to bind to the parts of the cell (i.e., ribosomes) where protein synthesis occurs.

In addition to reducing LH levels in the blood, alcohol may affect the activity of the LH molecule, rendering it less capable of stimulating hormone production in the testes. Like many other hormones, LH is not a simple protein but a protein to which various carbohydrates are attached (i.e., glycoprotein). The number and types of carbohydrates attached to the protein determine the hormone’s ability to stimulate testosterone production (i.e., its biological potency). Numerous LH variants exist with differing attached carbohydrates and differing potencies. Alcohol has been shown to result in the production of less potent LH molecules. Therefore, alcohol’s deleterious effects on LH function are qualitative as well as quantitative.

Whereas less information is available on alcohol’s effect on FSH, the secretion of this gonadotropin also appears to be reduced by alcohol. Alcohol does not appear to affect FSH synthesis, however (Emanuele et al. 1993).

## Alcohol, Opioids, and Reproduction

The opioid beta-endorphin is made in the hypothalamus as well as in other parts of the brain, in the pituitary, and in the testes. Hypothalamic beta-endorphin restrains the secretion of hypothalamic LHRH and thus is inhibitory to the HPG axis. Hypothalamic beta-endorphin increases with both acute and chronic alcohol exposure (Gianoulakis 1990), and increased beta-endorphin has been found to result in suppression of hypothalamic LHRH, pituitary LH, and testosterone synthesis, as noted previously. Alcohol-exposed animals and humans also have high levels of an estrogen known as estradiol. This is relevant, because estradiol is known to enhance the release of beta-endorphin. Thus, alcohol may increase opioids both directly and indirectly, although the magnitude of change is unknown.

Because opioids are known to play a role in oxidative injury, it is plausible that treatment with naltrexone, an opioid blocker, may prevent this injury. The use of naltrexone to accomplish such prevention is highly significant, as naltrexone is a drug already used clinically to reduce alcohol craving. As noted above, opioid blockers have been shown to help prevent alcohol-induced testosterone inhibition associated with opioids. Opioids also may play a role in the mediation of oxidative stress and apoptosis. The area of opioid blockade deserves broad attention.

## Effects of Paternal Alcohol Exposure on the Reproductive Axis

Few studies have addressed the effects of the male parent’s alcohol use on his reproductive ability and his offspring’s health (Bielawski and Abel 1997; Abel 1995). As a model of teenage drinking, researchers have studied the effects of alcohol exposure on peripubertal male rats. This research demonstrated the deleterious effects of paternal alcohol consumption on the offspring. Two months of alcohol feeding to male animals as they progressed through puberty resulted in their having lower body weights and reduced testosterone levels, compared with animals that did not receive alcohol. Despite this, after a 1-week abstinence from alcohol, those animals were able to successfully mate, although successful mating resulting in conception (i.e., fecundity) was significantly reduced and the number of successful pregnancies was diminished.

Although the litter size of the alcohol-exposed males was reduced by 46 percent, the average individual weight of the pup offspring of paternally alcohol-exposed animals was higher. The male-to-female pup ratio also was altered, with a higher preponderance of male offspring from alcohol-fed fathers. No gross malformations were noted among the pups.

Paternal testicular oxidative injury was increased, however, as demonstrated by enhanced lipid peroxidation. Germ cell apoptosis also was elevated in alcohol-exposed fathers (Emanuele et al. 2001). Therefore, it appears that chronic alcohol exposure in the peripubertal age group decreases fecundity, which may be mediated by testicular oxidative injury, leading to accelerated germ cell apoptosis in alcohol-exposed fathers. This is another exciting area for further investigation.

## Alcohol, Leptin, and Reproduction

Leptin, the hormone that regulates appetite discovered in 1994, has implications beyond its originally designated role. It seems to have a broad role in the HPG axis and beyond, for both men and women. In the HPG axis, leptin appears to stimulate LHRH and LH but inhibit gonadal activity (Hiney et al. 1999). In rats, 1 month of alcohol use stimulates leptin (Nicolas et al. 2001), presenting another potential and intriguing mechanism for alcohol-induced hypogonadism.

## Future Investigation

Research over the past 25 years has greatly expanded knowledge of alcohol’s effects on male reproduction. Areas fertile for investigation include mechanisms of alcohol-induced oxidative damage and apoptosis in the testes, the consequences of paternal alcohol exposure for their offspring, and the effects of alcohol use on leptin and male reproduction. In addition, practical prevention of testicular suppression with naltrexone and NO inhibition are promising therapeutic interventions.

## Footnotes

1 Oxidation reactions are those that remove hydrogen from a substance or add oxygen to it (or both).

2 Hydrogen atoms that are removed during oxidation are transferred to another molecule, which is thereby “reduced.” Thus, oxidation and reduction are complementary processes, because the oxidation of one substance entails the reduction of another. This process, which moves hydrogen atoms back and forth between oxidation–reduction reactions, helps to maintain an appropriate balance between oxidation and reduction in the cell.

## References

[b1-arcr-25-4-282] Abel EL (1995). A surprising effect of paternal alcohol treatment on rat fetuses. Alcohol.

[b2-arcr-25-4-282] Adams ML, Nock B, Truone R, Cicero TJ (1992). Nitric oxide control of steroidogenesis: Endocrine effects of N^G^-nitro-L-arginine and comparison to alcohol. Life Sciences.

[b3-arcr-25-4-282] Aleynik SI, Leo MA, Aleynik MK, Lieber CS (1998). Increased circulating products of lipid peroxidation in patients with alcoholic liver disease. Alcoholism: Clinical and Experimental Research.

[b4-arcr-25-4-282] Anderson RA, Quigg JM, Oswald C, Zaneveld LJ (1985). Demonstration of a functional blood-testis barrier to acetaldehyde. Evidence for lack of acetaldehyde effect on ethanol-induced depression of testosterone in vivo. Biochemical Pharmacology.

[b5-arcr-25-4-282] Azad N, Agrawal L, Emanuele MA, Kelley MR, Mohagheghpour N, Lawrence AM, Emanuele NV (1991). Neuroimmunoendocrinology. American Journal of Reproductive Immunology.

[b6-arcr-25-4-282] Berczi I, Nagy E, Kovacs K, Horwath E (1981). Regulation of humoral immunity in rats by pituitary hormones. Acta Endocrine.

[b7-arcr-25-4-282] Bielawski DM, Abel EL (1997). Acute treatment of paternal alcohol exposure produces malformations in offspring. Alcohol.

[b8-arcr-25-4-282] Chiao YB, Van Thiel DH (1983). Biochemical mechanisms that contribute to alcohol-induced hypogonadism in the male. Alcoholism: Clinical and Experimental Research.

[b9-arcr-25-4-282] Cicero TJ (1982). Alcohol-induced deficits in the hypothalamic-pituitary-luteinizing hormone axis in the male. Alcoholism: Clinical and Experimental Research.

[b10-arcr-25-4-282] Emanuele MA, Halloran MM, Uddin S, Tentler J, Emanuele NV, Lawrence AM, Zakhari S (1993). Effects of alcohol on the neuroendocrine control of reproduction. Alcohol and the Endocrine System: NIAAA Research Monograph No. 23.

[b11-arcr-25-4-282] Emanuele NV, LaPagli N, Steiner J, Colantoni A, Van Thiel DH, Emanuele MA (2001). Peripubertal paternal EtOH exposure. Endocrine.

[b12-arcr-25-4-282] Gianoulakis C (1990). Characterization of the effects of acute ethanol administration on the release of beta-endorphin peptides by the rat hypothalamus. European Journal of Pharmacology.

[b13-arcr-25-4-282] Gianoulakis C (1989). The effect of ethanol on the biosynthesis and regulation of opioid peptides. Experientia.

[b14-arcr-25-4-282] Hadley ME (1988). Endocrinology.

[b15-arcr-25-4-282] Hikim AP, Swerdloff RS (1999). Hormonal and genetic control of germ cell apoptosis in the testes. Reviews of Reproduction.

[b16-arcr-25-4-282] Hiney JK, Dearth RK, Lara F, Wood S, Srivastava V, Dees WL (1999). Effects of ethanol on leptin secretion and the leptin-induced luteinizing hormone (LH) release from late juvenile female rats. Alcoholism: Clinical and Experimental Research.

[b17-arcr-25-4-282] Jackson J, Klerekoper M (1990). Osteoporosis in men: Diagnosis, pathophysiology and prevention. Medicine.

[b18-arcr-25-4-282] Klein R, Duwall ES (1994). Bone loss in men: Pathogenesis and therapeutic considerations. Endocrinologist.

[b19-arcr-25-4-282] Lieber CS (1994). Hepatic and metabolic effects of ethanol: Pathogenesis and prevention. Annals of Medicine.

[b20-arcr-25-4-282] Little PJ, Adams ML, Cicero TJ (1992). Effects of alcohol on the hypothalamic-pituitary-gonadal axis in the developing male rat. Journal of Pharmacology and Experimental Therapeutics.

[b21-arcr-25-4-282] Nanji AA, Hiller-Sturmhöfel S (1997). Apoptosis and necrosis: Two types of cell death in alcoholic liver disease. Alcohol Health & Research World.

[b22-arcr-25-4-282] Nicolas JM, Fernandez-Sola J, Fatjo F, Casamitjana R, Bataller R, Sacanella E (2001). Increased circulating leptin levels in chronic alcoholism. Alcoholism: Clinical and Experimental Research.

[b23-arcr-25-4-282] Ogilvie KM, Rivier C (1997). Gender difference in hypothalamic-pituitary-adrenal axis response to alcohol in the rat: Activational role of gonadal steroids. Brain Research.

[b24-arcr-25-4-282] Polavarapu R, Spitz DR, Sim JE, Follansbee MH, Oberley LW, Rahemtulla A (1998). Increased lipid peroxidation and impaired antioxidant enzyme function is associated with pathological liver injury in experimental alcoholic liver disease in rats fed diets high in corn oil and fish oil. Hepatology.

[b25-arcr-25-4-282] Salonen I, Pakarinen P, Huhtaniemi I (1992). Effect of chronic ethanol diet in expression of gonadotropin genes in the male rat. Journal of Pharmacology and Experimental Therapeutics.

[b26-arcr-25-4-282] Sies H (1997). Oxidative stress: Oxidants and antioxidants. Experimental Physiology.

[b27-arcr-25-4-282] Stumble W, Thomas H, Stab W, Kuhn-Velveteen WN (1991). Tetrahydroisoquinoline alkaloids mimic direct but not receptor-mediated inhibitory effects of estrogens and phytoestrogens on testicular endocrine function. Possible significance for Leydig cell insufficiency in alcohol addiction. Life Sciences.

[b28-arcr-25-4-282] Vaubourdolle M, Guechot J, Chazouilleres O, Poupon RE, Giboudeau J (1991). Effect of dihydrotestosterone on the rate of ethanol elimination in healthy men. Alcoholism: Clinical and Experimental Research.

[b29-arcr-25-4-282] Yen SSC, Jaffe RB (1991). Reproductive Endocrinology.

[b30-arcr-25-4-282] Yin D, Mufson RA, Wang R, Shi Y (1999). Fas-mediated cell death promoted by opioids. Nature.

